# Radio-chimiothérapie adjuvante des adénocarcinomes gastriques: à propos de 34 cas et d'une revue de la literature

**DOI:** 10.11604/pamj.2014.19.70.5312

**Published:** 2014-09-24

**Authors:** Laurianne James, Sepos Dossou, Sarah Bellfiq, Joëlle Irigo, Etienne Ogandaga, Karima Mouden, Saïda Loughmari, Dounia Filali, Sanaa El Majjaoui, Tayeb Kebdani, Samir Ahid, Nourredine Benjafaar

**Affiliations:** 1 Service de Radiothérapie, Institut National d'Oncologie, CHU Ibn-Sina, Université Mohamed V, Rabat, Maroc; 2 Laboratoire de Biostatistique, de Recherche Clinique et d’Épidémiologie, Faculté de médecine et de pharmacie, Université Mohamed V, Rabat, Maroc; 3 Équipe de Recherche de Pharmaco-épidémiologie et Pharmaco-économie, Faculté de médecine et de pharmacie, Université Mohamed V, Rabat, Maroc

**Keywords:** Cancer gastrique, radio-chimiothérapie concomitante, adénocarcinomes, gastric cancer, concomitant chemoradiotherapy, adenocarcinomas

## Abstract

Notre étude consistera en l’évaluation du pronostic des patients porteurs d'un adénocarcinome gastrique opérés et traités par radio-chimiothérapie adjuvante en technique conformationnelle. Entre Janvier 2007 et Décembre 2011, 34 patients ont reçu après une chirurgie radicale (R0 ou R1), un à trois cycles de 5-Fluoro-uracile associé à de l'Elvorine en adjuvant, suivi d'une radio-chimiothérapie selon le même protocole à la dose de 45Gy, puis de deux cycles de chimiothérapie à un mois d'intervalle après la radio-chimiothérapie concomitante. Dans le groupe d’étude, il y avait 34 patients d’âge médian 50 ans (47-58), avec un sexe ratio (H/F) de 2,4. Une chirurgie de type R1 a été réalisée dans 26,5% des cas, et 53% des patients étaient de stade III-IV. Le rapport nombres de ganglions positifs, sur nombre de ganglions prélevés étaient > 0,4 dans 26,5% des cas. Durant le traitement mené à terme, une neutropénie de grade III a été observée chez quatre patients, avec des troubles digestifs (nausées, vomissement, ou diarrhée) de grade I/II dans la majorité des cas. Après un suivi médian de 20 mois, 70,6% des patients étaient en survie sans rechute, et 29,4% ont présenté une récidive métastatique; la survie globale à 5 ans était de 35,4% et la survie sans progression de 58,7%. La radio-chimiothérapie concomitante postopératoire pourrait être un régime efficace et sûre chez les patients ayant bénéficié d'une gastrectomie à visée curative dans le cancer de l'estomac localement avancé.

## Introduction

Le cancer gastrique est la 2^ème^ cause de décès lié au cancer chez l'homme et la quatrième chez la femme, représentant ainsi un véritable problème de santé dans le monde [1]. La maladie est souvent diagnostiquée à un stade localement avancé, et la chirurgie reste le principal traitement; Le taux de survie globale des patients ayant bénéficié seulement de la chirurgie est d'environ 45% à 5 ans et à subi peu de changements au cours de la dernière décennie. Les facteurs de risque indépendants relevés dans la littérature sont la taille tumorale (> 4 cm), l’âge (> 70 ans), la localisation proximale, le type diffus de la classification de Lauren, la présence d'un résidu tumoral, le degré d'invasion trans-pariétale (T3-T4), et le ratio ganglions envahis/nombre total prélevé (> 20%) [2, 3]. Les récidives locales sur le lit tumoral, sur l'anastomose, et dans les ganglions lymphatiques locorégionales surviennent chez 40 à 65% des patients après résection à visée curative. Pour palier à cela, une extension du geste chirurgicale à une splénectomie avec un curage ganglionnaire emportant au moins 25 ganglions a été préconisée sans que cela n'ai amélioré la survie, ni diminuer le taux de récidive locorégionale [4, 5]. Ainsi, pour les patients à haut risque de récidive de stade IB-IV sans métastases à distances, il est sans doute nécessaire qu'un traitement adjuvant soit proposé après la chirurgie [5, 6]. La plupart des essais adjuvants de radiothérapie ou de chimiothérapie n'ont pas démontré d'avantage sur la survie dans le cancer gastrique. Finalement l'intergroupe des cancers gastro-intestinaux (INT-0116) fut le premier à démontrer dans un essai de phase III que la radio-chimiothérapie concomitante après résection gastrique complète améliore la survie médiane sans rechute (30 vs 19 mois, p < 0,0001) et la survie globale (36 vs 27 mois, p < 0,01). Suite à ces résultats, la radio-chimiothérapie postopératoire selon le (protocole Macdonald) est devenue le nouveau standard. Cependant beaucoup de toxicités digestives demeurent de grade 3 (41%), et grade 4 (32%) liées à ce protocole [7]. L'objectif de cette étude est d’évaluation pronostic des patients porteurs d'un adéno-carcinome gastrique opérés et traités par radio-chimiothérapie adjuvante en technique conformationnelle

## Méthodes

Il s'agit d'une étude rétrospective de cancer gastrique traité selon le schéma décrit ci-dessous à l'Institut National d'Oncologie de Rabat entre Janvier 2007 et Décembre 2011. Les patients éligibles présentaient un adénocarcinome gastrique confirmé histologiquement sur une pièce de gastrectomie, de stade IB-IV (M0) selon le (Staging and Grouping System of American Joint Committee on Cancer AJCC/TNM 2002), et n'avaient bénéficié d'aucun autre traitement au préalable. Ils ont reçu un traitement chirurgical qui consistait en une gastrectomie totale, ou subtotale en fonction de la localisation tumorale. Les patients avec des marges saines (R0), ou un envahissement microscopique (R1) mais ne pouvant être repris chirurgicalement ont été inclus. Le curage ganglionnaire était orienté par le siège de la tumeur, en se basant sur les recommandations de la classification japonaise du cancer gastrique [6].

En postopératoire, le traitement comprenait une perfusion continue sur cinq jours de 5-fluoro-uracile 425mg/m^2^/j associée à de l'Elvorine 20mg/m^2^/j, puis 28 jours après, une radiothérapie de 45Gy. Deux cycles de chimiothérapie étaient administrés pendant la radiothérapie; un cycle pendant les quatre premiers jours de la radiothérapie avec 400mg/m^2^/j de 5-fluoro-uracile et 20mg/m^2^/j d'Elvorine, et une autre les 3 derniers jours de l'irradiation. Un mois après la fin de la radiothérapie, deux cycles de chimiothérapie à 28 jours d'intervalle étaient administrés selon le même protocole qu'avant la radiothérapie. La radiothérapie a été délivrée par un accélérateur linéaire d’énergie 6-18MV, à la dose de 45Gy en 25 fractions de 1,8Gy dans le volume cible prévisionnel (PTV), selon une technique conformationnelle à 3 ou 4 faisceaux. Le volume cible prévisionnel (PTV) correspondait au volume cible anatomo-clinique (CTV) et une marge circonférentielle 1,5 à 2 cm. Le volume cible anatomo-clinique englobait l'anastomose (oeso-jéjunale ou gastro-jéjunale), le moignon gastrique, le lit gastrique reconstruit à partir de la tomodensitométrie préopératoire associé au compte rendu endoscopique avec une marge de 2 cm, et les aires de drainages ganglionnaires. La limite inférieure du volume cible était fixée au niveau de la 3ème vertèbre lombaire (L3), dont l'objectif est d'inclure les ganglions sus et sous pylorique, coronaire stomachique, hépatique commun, tronc cœliaque, et ceux de l'artère hépatique. Les principaux organes à risque pris en compte étaient: le foie, les reins, la moelle, et l'intestin grêle.

Les patients étaient suivi de manière hebdomadaire en cours de traitement, afin d'apprécier la compliance au traitement, d’évaluer la perte de poids, et de rechercher les toxicités liés au traitement selon l’échelle CTC v.03 (Commun Toxicity Criteria de l'EORTC). L'efficacité thérapeutique était appréciée après le traitement, par un examen clinique complet, au besoin complété en fonction des signes cliniques par une fibroscopie oeso-gastrique ou par un examen radiologique (échographie abdominale, une radiographie thoracique +/- une tomodensitométrie thoraco-abdomino-pelvienne). Les patients étaient suivi tous les 3 mois au cours des 2 premières années, puis tous les 6 mois pendant 3 ans, et une fois par an au delà de la 5^ème^/ année. Les sites de récidives ont été classés ainsi: locorégional si récidive sur l'anastomose, carcinose péritonéale, ou métastases à distance (hépatique, pulmonaire, osseux). La survie globale (SG) a été définie comme le temps écoulé entre le traitement chirurgical et le décès, ou la dernière date à laquelle le patient a été vu vivant. La survie sans récidive (SSR) a été définie par le délai entre la chirurgie et la récidive du cancer (locale ou métastatique), ou la dernière date à laquelle le patient était connu indemne de la maladie

Nous avons utilisé pour l'analyse des statistiques, le logiciel de statistique pour les sciences sociales (SPSS) version 13.0. Les variables quantitatives ont été exprimées par la mesure de leur tendance (moyenne, médiane) et les mesures respectives de leur variabilité (écart type, quartile). La comparaison des caractéristiques cliniques et pathologiques s'est faite en utilisant le test T de Student, et de Mann-Whitney pour les variable quantitatives; pour les variables qualitatives par le test chi-square Pearson's ou le test exact de Fisher. La méthode de Kaplan - Meier a été utilisée pour estimer les taux de survie, et la comparaison des courbes a été réalisée par le test log-rank. Un intervalle de confiance à 95% avec un p < 0,05 était considéré comme statistiquement significatif.

## Résultats

Durant la période d’étude, 34 dossiers avaient répondu aux critères. La majorité était des hommes (70,6%), et l’âge médian était de 50 ans (47-58). Nous notions une prédominance de localisation distale (Antro-pylorique 55,9%), et l'ensemble des patients (76,6%) avait un performance status à 1. Le Tableau 1 ci-dessous présente les caractéristiques des patients répertoriées. Sur le plan histologique, tous les patients avaient un adénocarcinome dont dix neuf cas de composante en bague à chaton. La taille tumorale moyenne était de 5,75 +/- 2,4cm; il y avait 47% de patients de stade I-II, et 53% de cas pour le stade III-IV. Le Tableau 2 ci-après présente les caractéristiques de la tumeur. Le délai entre la chirurgie et la chimiothérapie d'induction était de 77 jours (47-117); le taux d'hémoglobine avant la chimiothérapie était de 11,7g/dl (10-12,85), et 14 patients (41,2%) ont reçu 2-3 cures de chimiothérapie avant la radio-chimiothérapie. Le nombre médian de cures de chimiothérapie pour l'ensemble des patients était de 4 cycles [3–5]. Cette chimiothérapie a été à l'origine d'une neutropénie de grade III chez 4 patients, et de toxicités digestives à type de vomissements (55,9%), et diarrhées de grade I-III (68,8%), résumées dans le Tableau 3 joint ci-dessous. La radiothérapie a débuté après un intervalle médian de 47 jours (33-117). Le traitement a été mené à son terme chez tous les patients, avec cependant des toxicités digestives de GI-II, comme décrit dans le Tableau 3 ci-dessus. Seul 8,8% des patients ont présenté une neutropénie de grade III. La durée médiane de l’étalement de la radiothérapie a été de 42 jours (37-51). A distance, aucune toxicité tardive n'a été retrouvée.

**Tableau 1:** Caractéristiques des patients répertoriés pour l’étude

Caractéristiques	Valeur N (%)
Age*	50 (47- 58)
Sexe: Masculin	24 (70,6%)
Performance status	
PS 0	10 (29,4%)
PS 1	24 (70,6%)
**Localisation tumorale**	
Cardia-Fundus	5 (14,7%)
Corps	10 (29,4%)
Antro-pylorique	19 (55,9%)
**Type de chirurgie**	
Gastrectomie totale	16 (47,1%)
Gastrectomie partielle	18 (52,9%)
**Différenciation histologique de l'ADK**	
Bien différencié	6 (17,6%)
Moyennement différencié	16 (47,1%)
Peu différencié	12 (35,3%)
Etat des marges**	
R0	25 (73,5%)
R1	9 (26,5%)
Curage ganglionnaire***	
D0	21 (61,8%)
D1	13(38,2%)
Taux hémoglobine (g/dl) avant RCC*	11,7 (10-12,8)

* Les valeurs sont exprimées en médiane(quartile).

** Le statut des marges: R0 pour non tumoral, R1 pour envahissement microscopique.

*** Le curage ganglionnaire: D0 < 15 ganglions et, D1 ^3^ 15 ganglions prélevés.

**Tableau 2:** Caractéristique histologique postopératoire des pièces de gastrectomie

Caractéristiques	Valeur N (%)
Taille tumorale*	5,75 ± 2,4
pT**	
T2	16 (47, 1%)
T3	18 (52, 9%)
pN**	
N0	4 (11, 8%)
N1	9 (26,5%)
N2	14 (41, 2%)
N3	7 (20, 6%)
**Stades**	
IB	1(2, 9%)
II	15 (44, 1%)
IIIA	12 (35, 4%)
IIIB	3 (8, 8%)
IV	3 (8, 8%)
Ratio N + /N total***	
< 0,4	25 (73, 5%)
^3^ 0,4	9 (26, 5%)
**Embole vasculaire**	
Oui	13 (38, 2%)
Non	21 (61, 8%)

* Les valeurs sont exprimées en moyenne ± écart type.

** Le pT, pN représente la classification postopératoire des tumeurs gastrique, pT (Tumeur) et pN (ganglion envahi).

*** Le ratio N + /N total représente le rapport nombre de ganglion envahit sur le nombre total de ganglion prélevé.

**Tableau 3:** Toxicité de la chimiothérapie et de la radio-chimiothérapie selon le CTC v.03 de l'EORTC

Type de toxicité	Chimiothérapie			Total N (%)	Radio-chimiothérapie			Total N (%)
	Grade				Grade			
	GI	GII	GIII		GI	GII	GIII	
Epigastralgie					7	2		9 (26, 5%)
Diarrhée	8	11	1	20(68, 8%)	14	10	1	25 (73, 5%)
Vomissement	11	7	1	19(55, 9%)	5	13		18 (52, 9%)
Neutropénie		1	4	5(14, 7%)			3	3 (8, 8%)

**Figure 1:**
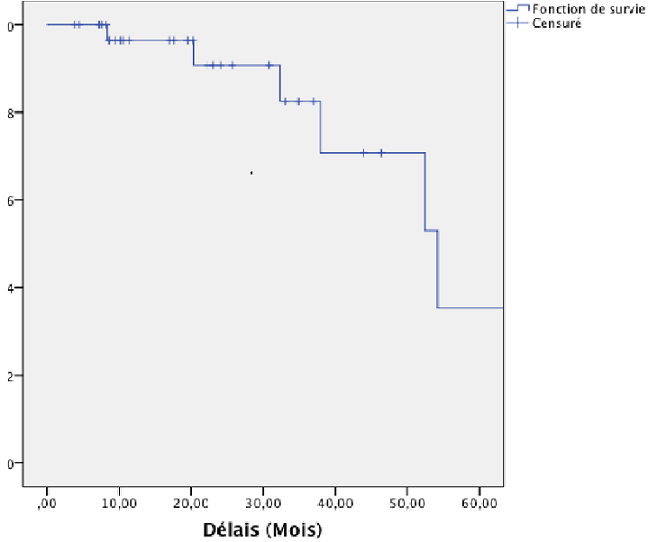
Survie globale de l'ensemble des patients à 5 ans selon Kaplan-Meier qu'ils aient récidivé ou présenté des metastases

**Figure 2:**
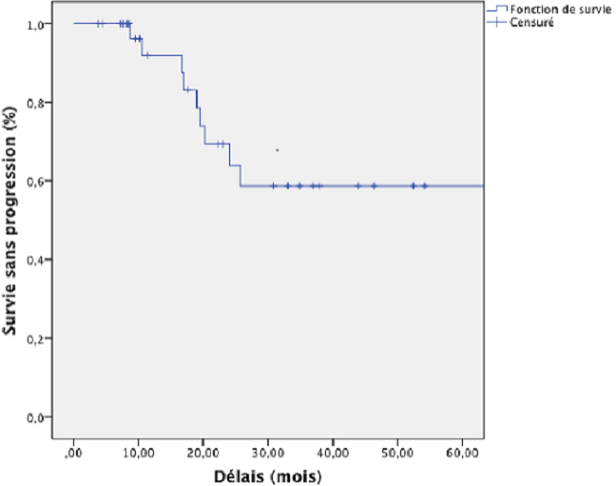
Survie sans progression de l'ensemble des patients à 5 ans selon Kaplan-Meier quelque soit de l’état des marges chirurgicales (R1/R0)

Concernant les facteurs de risque, il y avait plus de récidive chez les patients qui avaient des marges positives, par rapport à ceux ayant des marges négatifs et, cette différence était statistiquement significatif (p=0,009). Les patients avec un curage D0 avaient un risque de décès plus élevé que ceux ayant un curage D1, et cette différence est statistiquement significative (p= 0,03). La population d’étude à eu un suivi médian de 20 mois, la progression a été rapportée chez 10 patients (29,4%) dont, deux ont présenté des récidives locorégionales (5,9%), et 8 des métastases à distance (23,5%), avec une survie globale à 5 ans de 35,4% représentée sur le Figure 1. Le site préférentiel des métastases à distance était dans 17,6% au niveau pulmonaire, 11,8% de carcinose péritonéale, 5,9% de métastases hépatiques, et 2,9% de métastases osseuses. La survie sans progression à 5 ans était de 58,7% représentée sur le Figure 2, et la positivité des marges à eu un impact négatif sur la survie sans récidive et cette différence était statistiquement significative p=0,0001.

## Discussion

Sur les 34 dossiers analysés, 26,5% ont eu une chirurgie de type R1, et 38,2% un curage D1. Après la chirurgie, 44,1% des patients étaient de stade II, et 52% de stade III-IV; 52,9% des patients ont présenté des toxicités digestives à type de vomissement de grade I-II, et une diarrhée de grade I-III (73,5%) au cours de la radio-chimiothérapie concomitante. Après un suivi médian de 20 mois, 29,4% ont présenté des récidives dont 5,9% locorégionales, et 23,5% de métastases à distances. La survie sans progression à 5 ans était de 58,7% et la survie globale de 35,4%.

Le cancer gastrique est souvent diagnostiqué lorsqu'il est localement évolué. La gastrectomie associée à un curage ganglionnaire en fonction de la topographie lésionnelle, est le traitement de référence. Cependant, le taux de contrôle et de survie à 5 ans reste faible, de l'ordre de 20-30% chez les patients T3-T4, avec des ganglions positifs [8]. Cette constatation a incité non seulement, les chirurgiens à élaborer de nouvelles approches chirurgicales avec un curage ganglionnaire étendu mais également, elle a été à l'origine de plusieurs études sur les possibilités thérapeutiques post-opératoires. Ainsi, l’étude américaine du Southwestern oncology group (SWOG)/intergroup 0116 publiée en 2001, à comparé une chirurgie seule, suivie ou non d'une chimio-radiothérapie [9]; la population étudiée était composée de tumeurs T3 dans plus de 60% des cas, avec un envahissement ganglionnaire dans 85% des cas. A 3 ans, les risques relatifs de récidive et de décès en l'absence de traitement adjuvant étaient augmentés respectivement de 52% (hazard ratio: 1,52; IC95%: 1,23-1,86) et 35% (hazard ratio: 1,35; IC95%: 1,09-1,66). Le taux de survie sans récidive a trois ans était de 41% dans le bras chirurgie seule, et de 50% dans le bras chirurgie suivit d'une radio-chimiothérapie (p< 0,001); de même, la survie globale à trois ans était de 41% dans le bras chirurgie seule, contre 48% dans celui suivit d'une radiothérapie (p=0,005). L'analyse de la courbe de survie du bras combiné montre que le taux de survie à 5 ans était de l'ordre de 30%. Ces résultats ont été confirmés par d'autres auteurs qui ont rapporté un bénéfice sur la survie globale avec la radio-chimiothérapie postopératoire chez des patients présentant un cancer gastrique avec curage ganglionnaire insuffisant ou lorsqu'il s'agit d'une tumeur T3-T4 et/ou N+ [10, 11]. Dans notre étude, tous les patients ont reçu une radio-chimiothérapie postopératoire, et le traitement a été mené a terme, avec une survie globale à 5 ans de 35,4% (médiane de survie de 54,17 mois). Ces résultats sont comparables à ceux retrouvés par l'INT 0116, et par Bleiberg et al [12] qui a eu une survie globale à 5 ans de 33%. Dans l’étude de l'INT-0116, sur les 281 patients randomisés pour recevoir la radio-chimiothérapie concomitante, seuls 181 (64%) ont terminé leur traitement en raison des toxicités de grade 3-4 survenues chez 41% et 32% des patients. De même Lim et al [13] on rapporté des toxicités gastro-intestinales (nausées-vomissements, et diarrhées) de grade 3-4 dans 10-12% des cas. Selon les données répertoriées dans les dossiers des patients de notre étude, en se basant sur les critères de toxicités de l'EORTC, quatre patients ont présenté une neutropénie de grade III, et dans 3 cas (8,8%) une réduction des doses de chimiothérapie a été fait à la 4^ème^ cure; des troubles digestifs à types de diarrhée, et de vomissement de grade I-II ont été rapporté dans 52,9% des cas probablement du au fait que la radiothérapie réalisée était conformationnelle.

Trois études contrôlées ont comparé une gastrectomie totale, à une gastrectomie subtotale; les résultats ont été rapportés en termes de morbidité et de mortalité, puis secondairement en termes de survie. Il n'y avait pas de différence en termes de mortalité iatrogène entre les deux groupes 1,3% et 3,2%, avec un taux de survie à 5 ans de 48% dans les deux bras [14]. De même, dans notre étude 47,1% des patients ont eu une gastrectomie totale, et 52,9% une gastrectomie partielle sans aucune différence sur le taux de survie à 5 ans 64% et 49,5% respectivement (p = 0,29). Le bénéfice en survie globale, et en survie sans récidive dans l'essai de l'INT0116 a été obtenu alors que 54% des patients avaient eu un curage D1. Rétrospectivement, l'indice de Maruyama qui permet d'estimer la probabilité d'envahissement des stations ganglionnaires de la classification japonaise à partir des données cliniques et anatomopathologique a été calculé dans la population d’étude de l'INT 0116; l'indice moyen très élevé retrouvé dans la population américaine signifiait qu'une proportion très importante, estimée à 70% des patients, avait eu une résection incomplète, alors que le protocole demandait une résection D2. Cependant, l’écart entre la chirurgie demandée et celle réalisée reflétait la réalité de la pratique chirurgicale aux Etats-Unis à la fin des années 1990 [15, 16]. Dans notre contexte, vu que le traitement chirurgical a été effectué chez la plupart des patients dans d'autres structures hospitalières seul 38,2% des patients ont eu un curage D1 et 61,8% un curage D0 avec un impact à la limite de la significativité sur la survie globale (p = 0,05) mais pas sur la survie sans récidive (p= 0,97). Selon l'American joint committee on cancer (AJCC), le nombre de ganglions positifs retrouvé est un facteur de mauvais pronostic sur la survie. En effet, selon certains auteurs le nombre de ganglions et la présence d'emboles vasculaires sont des facteurs influençant négativement la survie des patients atteints d'un cancer gastrique [17–19]. Marchet et al [19] ont montré qu'il y avait une corrélation entre le nombre de ganglions prélevés (N < 16) et la survie globale qui était plus faible comparée à celle des patients ayant un curage comportant plus de 16 ganglions. Wydmanski [20] a reporté que le ratio nombre de ganglions positifs sur le nombre de ganglion total (< 0,6) était lié a un meilleur pronostic. Dans la présente étude, le ratio nombre de ganglions positifs sur le nombre total de ganglions prélevés (< 0,4) n'a pas eu un impact sur la survie globale (p = 0,76); on constate cependant, que la qualité de la chirurgie (marge R1), diminue significativement la survie sans progression (p < 0,0001), ce résultat est comparable à ceux retrouvés dans la plupart des études rétrospectives et prospectives [21, 22].

Selon les recommandations de la Société Européenne d'Oncologie Médicale (ESMO) publiées en 2013, les patients avec une chirurgie de type R1 bénéficient d'un traitement palliatif [23]; Bien que la résection R1 soit considérée comme un facteur pronostic défavorable, elle ne devrait pas disqualifier ces patients pour lesquels la survie médiane après chirurgie seule n'est que de 15%. A ce jour, les séquences thérapeutiques (chirurgie, radiothérapie, et chimiothérapie) sont incertaines, mais pourront faire l'objet de sujet des futurs essais. Les autres facteurs de risque (âge, taille tumoral, le degré de différenciation) connus pour leur impact négatif sur la survie n'ont pas été retrouvé; cela peut s'expliquer par le faible effectif de notre étude (34 cas). Après un suivi médian de 20 mois, dix patients (29,4%) ont présenté des récidives locorégionales et à distance; ces résultats sont proches de ceux des autres séries 29-50% [9, 13, 20, 24]. Les limites de notre étude sont liées d'une part à notre faible effectif, du au fait que la majorité des patients proviennent d'autres structures; et d'autre part, à l'absence de données concernant les toxicités tardives liés à la radio-chimiothérapie concomitante. Indépendamment de cela, la faisabilité du protocole de radio-chimiothérapie concomitante selon ce schéma thérapeutique a permis d'obtenir une durée médiane de survie de 54,17 mois.

## Conclusion

Le pronostic défavorable des cancers de l'estomac localement évolués a justifié la réalisation de nombreux essais évaluant les traitements adjuvants à la chirurgie. La radio-chimiothérapie a été évaluée dans un large essai contrôlé qui a montré une amélioration significative des taux de survie globale et de survie sans récidive après une gastrectomie pour des cancers de stade IB-IV. Cependant, le protocole de chimiothérapie était responsable de nombreuses toxicités digestives et hématologiques de grade III-IV. Bien que notre étude ai un effectif faible, ce protocole est réalisable dans notre institut avec une assez bonne tolérance, et sans interruption du traitement; les décès étaient soit en rapport avec des récidives locorégionales ou des métastases et seule la qualité de la chirurgie (marge R0/R1) a été notre principal facteur pronostic de récidive après la radio-chimiothérapie concomitante.

## References

[1] Garcia M, Ward EM, Center MM, Hao Y, Siegel RL, Thun MJ (2007). Global Cancer Facts and Figures. http://www.cancer.gov.

[2] Kim JP, Kim YW, Yang HG, Noh DY (1994). Significant prognostic factors by multivariate analysis of 3926 gastric cancer patients. World J Surg..

[3] Kajiyama Y, Tsurumaru M, Udagawa H, Tsutsumi K, Kinoshita Y, Ueno M (1997). Prognosticfactors in adenocarcinoma of the gastric cardia: pathologic stage analysis and multivariate regression analysis. J Clin Oncol..

[4] Gunderson LL (2002). Gastric cancer - patterns of relapses aftersurgicalresection. Semin Radiat Oncol..

[5] Mc Culloch P, Nita ME, Kazi H, Gama-Rodrigues J (2003). Extended versus limited lymphnodes dissection technique for adenocarcinoma of the stomach. Cochrane Database SystRev.

[6] Landry J, Tepper JE, Wood WC, Moulton EO, Koerner F, Sullinger J (1990). Patterns of failure following curative resection of gastric cancer. Int J Radiat Oncol BiolPhys..

[7] Macdonald JS, Smalley SR, Benedetti J, Hundahl SA, Estes NC, Stemmermann GN (2001). Chemoradiotherapy after surgery compared with surgery alone for adenocarcinoma of the stomach or gastroesophageal junction. N Engl J Med..

[8] Landry J, Tepper JE, Wood WC (1990). Analysis of survival and local control following surgery for gastric cancer. Int J RadiatOncolBiolPhys..

[9] Macdonald JS, Smalley S, Benedetti J (2004). SWOG; ECOG; RTOG; CALGB; NCCTG. Postoperative combined radiation and chemotherapy improves disease-free survival (DFS) and overall survival (OS) in resected adenocarcinoma of the stomach and GE junction: update of the results of intergroup study INT-0116 (SWOG 9008).

[10] Kim S, Lim DH, Lee J, Kang WK, MacDonald JS, Park CH (2005). An observational study suggesting clinical benefit for adjuvant postoperative chemoradiation in a population of over 500 cases after gastric resection with D2 nodal dissection for adeno-carcinoma of the stomach. Int J Radiat Oncol Biol Phys..

[11] Bora H, Unsal D, Akmansu M (2004). Results of chemoirradiation after curative resection of locally advanced gastric cancer. Int J Clin Pract..

[12] Bleiberg H, Goffin JC, Dalesio O, Buyse M, Pector JC, Gignoux M (1989). Adjuvant radiotherapy and chemotherapy in resectablegastric cancer. A randomized trial of the gastro-intestinal tract cancer cooperative group of the EORTC. Eur J SurgOncol..

[13] Lim DH, Kim DY, Kang MK, Kim YI, Kang WK, Park CK (2004). Patterns of failure in gastric carcinoma after D2 gastrectomy and chemoradiotherapy: a radiation oncologist's view. Br J Cancer..

[14] Gouzi JL, Huguier M, Fagniez PL, Launois B, Flamant Y, Lacaine F (1989). Total versus subtotal gastrectomy for adenocarcinoma of the gastric antrum: a french prospective controlledstudy. Ann Surg..

[15] Bollschweiler E, Boettcher K, Hoelscher AH, Sasako M, Kinoshita T, Maruyama K (1992). Preoperative assessment of lymphnode metastases in patients with gastric cancer: evaluation of the Maruyama computer program. Br J Surg..

[16] Guadagni S, de Manzoni G, Catarci M, Valenti M, Amicucci G, De Bernardinis G (2000). Evaluation of the Maruyama computer program accuracy for preoperative estimation of lymphnode metastases from gastric cancer. World J Surg..

[17] Bora H, Unsal D, Akmansu M (2004). Results of chemoirradiation after curative resection of locally advanced gastric cancer. Int J Clin Pract..

[18] Kattan MW, Karpeh MS, Mazumdar M, Brennan MF (2003). Postoperative nomogram for disease - specific survival after an R0 resection for gastric carcinoma. J Clin Oncol..

[19] Marchet A, Mocellin S, Ambrosi A, Morgagni P, Garcea D, Marrelli D (2007). Italian Research Group for Gastric Cancer (IRGGC): The ratio between metastatic and examined lymphnodes (N ratio) is an independent prognostic factor in gastric cancer regardless of the type of lymphadenectomy: results from an Italian multicentric study in 1853 patients. Ann Surg..

[20] Wydmanski J (2008). Study of effectiveness and tolerance of pre- and postoperative radiochemotherapy for patients with stomach cancer. Nowotwory J Oncol..

[21] Hermanek P, Wittekind C (1994). Residual tumor (R) classification and prognosis. Min Surg Oncol..

[22] Marrelli D, De Stefano A, de Manzoni G, Morgagni P, Di Leo A, Roviello F (2005). Prediction of recurrence after radical surgery for gastric cancer: a scoring system obtained from a prospective multicenter study. Ann Surg..

[23] Jackson C, Cunningham D, Oliveira J, ESMO Guidelines Working Group (2009). Gastric cancer: ESMO clinical recommendations for diagnosis, treatment and follow-up. Ann Oncol..

[24] Maehara Y, Hasuda S, Koga T, Tokunaga E, Kakeji Y, Sugimachi K (2000). Post- operative outcome and sites of recurrence in patients following curative resection of gastric cancer. Br J Surg..

